# Estimated Oral Semaglutide Exposure Has Distinct Relationships With Glycaemic Response, Weight Loss and Gastrointestinal Tolerability

**DOI:** 10.1111/dom.70840

**Published:** 2026-05-10

**Authors:** Gian Paolo Fadini, Mario Luca Morieri, Carlotta Boscaro, Benedetta Maria Bonora, Andrea Cignarella

**Affiliations:** ^1^ Department of Medicine University of Padova Padua Italy; ^2^ Veneto Institute of Molecular Medicine Padua Italy

**Keywords:** pharmacokinetics, real‐world evidence, semaglutide, type 2 diabetes

## Abstract

**Aims:**

Oral semaglutide absorption is subject to inter‐individual variability. We investigated whether estimated individual exposure (*eC*_*avg*_) provides predictive information beyond the prescribed dose in a real‐world cohort of patients with type 2 diabetes (T2D).

**Materials and Methods:**

We retrospectively included patients initiating oral semaglutide to calculate *eC*_*avg*_ at each follow‐up visit, using a validated population pharmacokinetic model incorporating dose, weight, sex, ethnicity and GI disorders, but without measuring plasma semaglutide concentrations. Mixed‐effects models were used to assess the associations of dose and *eC*_*avg*_ with changes in HbA1c and percent body weight, and to compare model fit. For GI side effects, time‐adjusted fixed‐effects logistic regression was employed.

**Results:**

We analysed 256 participants (31.6% women, mean age 65.6 years). During a median follow‐up of 19 months, HbA1c declined by 0.7% and body weight by 7.5%; 41.8% of patients reported GI side effects. Both dose and *eC*_*avg*_ were significantly associated with all outcomes when analysed individually. For ΔHbA1c, the dose model outperformed the *eC*_*avg*_ model (*p* < 0.01), while *eC*_*avg*_ showed modestly improved fit for weight loss (*p* < 0.001). The exposure‐effect relationship was clearly right‐shifted for weight loss compared to the glycaemic effect. For GI side effects, *eC*_*avg*_ remained significantly associated with the outcome after adjustment for dose residuals, indicating that *eC*_*avg*_ had greater predictive value for tolerability.

**Conclusions:**

Although estimated and not measured, the exposure to oral semaglutide may provide outcome‐dependent information. While the prescribed dose seems sufficient to predict glycaemic response, estimated exposure provides additional value for weight loss and GI tolerability.

## 1 Introduction

Glucagon‐like peptide‐1 receptor agonists (GLP‐1RAs) have become foundational for the management of type 2 diabetes (T2D), due to their robust glycaemic efficacy, favourable effects on body weight and cardiovascular benefits [1]. Among these, semaglutide is available as both a subcutaneous injection and an oral formulation, the latter being made possible by the absorption enhancer sodium N‐(8‐[2‐hydroxybenzoyl] amino) caprylate (SNAC), which facilitates transcellular absorption in the stomach [2, 3].

Efficacy of oral semaglutide has been proven in various patient subgroups in the PIONEER trial programme [4] and has been shown to be similar to that of the once‐weekly injectable formulation under real‐life conditions [5]. However, there is considerable inter‐individual variability in its absorption and, consequently, in the resulting plasma concentrations [6]. This variability is influenced by factors such as body weight, sex and the presence of gastrointestinal conditions, and may have important implications for the effectiveness of treatment under routine care [7].

Gastrointestinal side effects, most notably nausea and vomiting, remain a major challenge with GLP‐1RA therapy [8]. These adverse events can affect treatment persistence and may limit the ability to up‐titrate to most effective doses [9]. Nauck and colleagues have recently highlighted that the development of tolerance to these side effects is closely related to the characteristics of the dose‐escalation regimen, with longer, multi‐step titration schedules being associated with better tolerability and greater therapeutic efficacy [10].

The key distinction between the prescribed dose and the actual exposure achieved in an individual patient is often overlooked in clinical pharmacology. While dose is a simple, easily recorded metric, the resulting plasma concentration integrates inter‐individual differences in absorption, distribution and clearance. Overgaard et al. [7] have previously shown, using data from the PIONEER and SUSTAIN trials, that the circulating level of semaglutide is the primary determinant of reductions in both HbA1c and body weight, irrespective of the route of administration. They also developed a simple estimate of exposure (*eC*_*avg*_) derived from dose and routinely available clinical data.

The present study was designed to investigate whether *eC*_*avg*_ provides predictive information beyond the prescribed dose for three key outcomes in a real‐world cohort of patients with T2D initiating oral semaglutide: (i) glycaemic control (ΔHbA1c), (ii) weight loss (percent Δbody weight) and (iii) gastrointestinal tolerability (proportion with GI side effects). We aimed to formally test the added value of estimated pharmacokinetics over the simple prescribed dose in routine clinical practice.

## 2 Methods

### 2.1 Study Design and Participants

This was a retrospective study conducted at the diabetes outpatient clinic of the University Hospital of Padua. We identified and included all male or female participants aged 18–90 years with a diagnosis of T2D, who initiated for the first time oral semaglutide without being previously treated with a GLP‐1RA (naïve). Patients were excluded if they did not satisfy a T2D diagnosis, if age was < 18 or > 90 years, if they were switching from another GLP‐1RA, and if HbA1c was < 6.5%. There were no inclusion/exclusion criteria based on BMI. The study was approved by the local ethical committee and conducted in accordance with the declaration of Helsinki. In agreement with the national regulation on retrospective studies on anonymised data, the need for informed consent was waived.

### 2.2 Variables

For all patients, we recorded the following variables with the scope of defining the population characteristics: demographics (age, sex, ethnicity and diabetes duration); anthropometrics (height, weight, BMI); risk factors (smoking, hypertension and dyslipidaemia); laboratory exams (fasting glucose, HbA1c, lipids, serum creatinine for the calculation of eGFR and urinary albumin excretion ratio [UACR]); complications and medications, as previously described [11].

### 2.3 Definition of Exposure

At each visit, we recorded the prescribed dose of oral semaglutide (3 mg, 7 mg, 14 mg) and whether the drug was continued to be prescribed. No information was available on pharmacy refill rates and adherence. Plasma semaglutide concentrations were not measured, as retrospective real‐world studies do not include prospectively collected and stored plasma samples that would be required for pharmacokinetic analyses. We calculated the estimated average exposure under steady‐state assumptions (eC_avg_) as previously described [7] from the following covariates: prescribed dose, body weight, sex, Asian ethnicity, presence of upper GI tract disorders. The final equation, based on the pharmacokinetic model described in [7], was as follows: *eC*_*avg*_ = 1.2233*dose (mg) *(0.814^male sex (1/0))*(1.1^Asian (1/0))*(1.13^upper GI tract disorders (1/0))*((body weight / 85)^−0.636). For each follow‐up visit, *eC*_*avg*_ was calculated using the prescribed dose and the updated body weight. Therefore, dose escalation, dose maintenance, or failure to escalate were reflected in the visit‐level exposure estimate.

### 2.4 Outcomes of Interest

The index date was set as the date of first oral semaglutide prescription. At each subsequent visit, we recorded updated values of HbA1c and body weight to calculate glycaemic efficacy (ΔHbA1c) and weight loss from baseline. At each time point, we also recorded whether the patient experienced GI side effects mapping the following MEDDRA terms, as typically done in clinical trials [12]: nausea, vomiting, GI complaints, diarrhoea, constipation, dyspepsia, abdominal pain.

### 2.5 Statistical Analyses

Continuous data are presented as mean and standard deviation, while categorical data are presented as number and percentage. Correlations were analysed with Pearson's coefficients. To evaluate the relationship between drug exposure and clinical efficacy, an exposure‐response analysis was conducted for changes in HbA1c and percent body weight using *eC*_*avg*_ and longitudinal data collected at multiple time points. For each patient, the change from baseline in HbA1c and the percentage change in body weight were plotted against the corresponding *eC*_*avg*_ values. The relationship was described using a nonlinear mixed‐effects modelling approach, consistent with the standard *E*_*max*_ pharmacodynamic model. The model was parameterised to estimate the maximum achievable effect (*E*_*max*_) and the concentration producing half‐maximal effect (*EC*_*50*_). Goodness of fit was assessed by visual inspection of the model‐predicted curves and their 95% confidence bands relative to the observed data.

In order to characterise the relationship between drug exposure and safety, an exposure‐response analysis was performed for GI side effects using individual *eC*_*avg*_ and longitudinal binary outcome data. The probability of experiencing an event was modelled as a function of *eC*_*avg*_ using a logistic regression. For descriptive purposes, a four‐parameter logistic curve was fitted to the aggregated data to visualise the exposure‐response relationship, with the bottom and top constrained to 0 and 1. Goodness of fit was assessed visually from the plotted curve and its 95% confidence bands.

To compare the predictive performance of dose and *eC*_*avg*_, we ran mixed‐effects models including either dose or *eC*_*avg*_ as the exposure metric. Due to the high co‐linearity between dose and *eC*_*avg*_, they were not included simultaneously in the same model. To further explore their independent contributions, we fitted an additional model including *eC*_*avg*_ together with dose residuals. Model performance was compared using the delta −2log likelihood ratio test (the lower the −2LL, the better the model fits observed data). We also computed the correlation coefficients between observed data (change in HbA1c and weight) and those predicted by the respective models. Correlation coefficients were compared using the Fisher transform method.

To obtain pure within‐subject estimates for the occurrence of GI side effects, and to control for all unmeasured time‐invariant confounders, we used a fixed‐effects logistic regression model (i.e., a model including patient identifier as a categorical predictor). All models were adjusted for the baseline value of the respective outcome (HbA1c or weight) to account for regression to the mean. To model GI side effects, time (in months) was also included as a covariate in the fixed‐effects models. No additional covariates were included, as the primary aim was to compare the predictive value of *eC*_*avg*_ versus prescribed dose. SPSS version 28 or later was used, and statistical significance was accepted at the conventional level of *p* < 0.05.

## 3 Results

### 3.1 Patient Characteristics

We included 256 new‐users of oral semaglutide, naïve to GLP‐1RA therapy. They were 31.5% female with a mean age of 65 years and a mean diabetes duration of 12.6 years. Baseline BMI was 29.3 kg/m^2^, and HbA1c was 8.0%. Most patients (85.9%) were receiving metformin and the second most common concomitant oral agents were SGLT2i (37.9%), while only 14.8% were also receiving insulin. Other clinical characteristics are reported in Table 1.

**TABLE 1:** Baseline characteristics of study participants. Data are presented as mean (standard deviation) for continuous variables, while categorical variables are presented as number (%). UACR, urinary albumin creatinine ratio. RAS, renin angiotensin system.

Clinical variable	All participants
Demographics	*N* = 256
Female, *n* (%)	81 (31.6)
Age, years	65.6 (11.7)
Asian, *n* (%)	16 (6.3)
Diabetes duration, years	12.6 (8.8)
Risk factors and laboratory	
Body mass index, kg/m^2^	29.3 (5.4)
Weight, kg	84.6 (16.8)
Current smoking, *n* (%)	22 (8.6)
Hypertension, *n* (%)	215 (84.0)
Systolic blood pressure, mm Hg	145.9 (20.2)
Diastolic blood pressure, mm Hg	79.5 (11.7)
Fasting glucose, mg/dl	164.2 (57.4)
HbA1c, %	8.0 (1.3)
Total cholesterol, mg/dl	157.0 (42.8)
HDL cholesterol, mg/dl	47.6 (11.6)
Triglycerides, mg/dl	152.9 (123.5)
LDL cholesterol, mg/dl	78.1 (33.5)
eGFR, ml/min/1.73 m^2^	81.5 (21.9)
UACR, mg/g	114.5 (531.1)
Complications	
Normo‐albuminuria, *n* (%)	145 (56.6)
Micro‐albuminuria, *n* (%)	54 (21.1)
Macro‐albuminuria, *n* (%)	10 (3.9)
Retinopathy, *n* (%)	62 (24.2)
Neuropathy, *n* (%)	45 (17.6)
Cardiovascular disease, *n* (%)	49 (19.1)
Coronary artery disease, *n* (%)	49 (19.1)
Peripheral arterial disease, *n* (%)	32 (12.5)
Therapies	
Metformin, *n* (%)	220 (85.9)
SGLT2 inhibitors, *n* (%)	97 (37.9)
Sulphonylureas, *n* (%)	21 (8.2)
Pioglitazone, *n* (%)	9 (3.5)
Insulin, *n* (%)	38 (14.8)
RAS blockers, *n* (%)	150 (58.6)
Statins, *n* (%)	214 (83.6)

### 3.2 Follow‐Up and Overall Effectiveness

**FIGURE 1:**
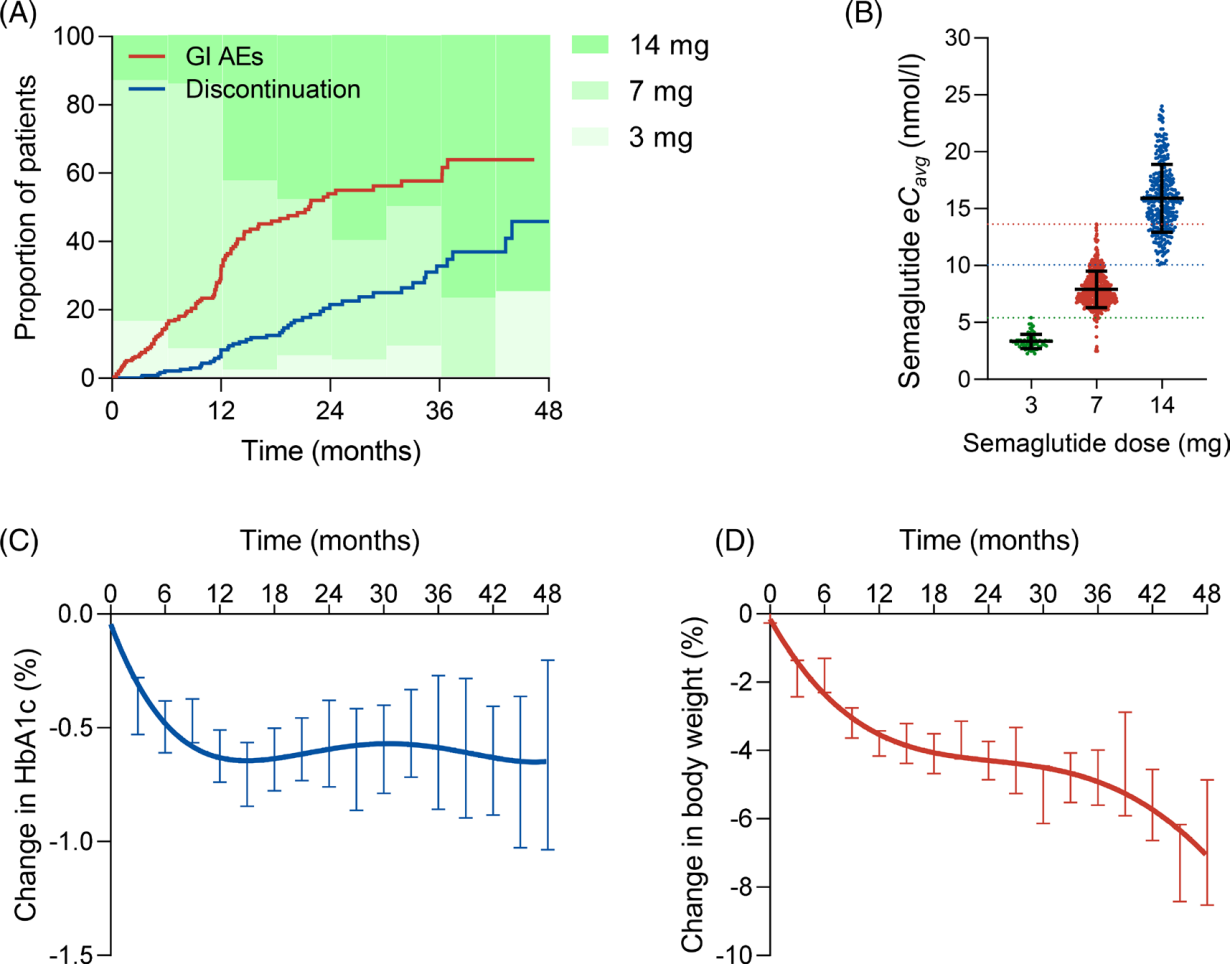
Observed effects of oral semaglutide. (A) Proportion of participants who experienced gastrointestinal adverse events (GI AEs) or discontinued treatment over time. The background green colours indicate the various doses during the observation. (B) Estimated average concentration (*eC*_*avg*_) or oral semaglutide (exposure) versus prescribed dose (horizontal dotted lines highlight the overlap in the achieved concentrations). (C, D) Observed change in HbA1c (C) and percent body weight (D) analysed with a mixed model for repeated measures (bars indicate 95% confidence interval, while the line is interpolated using a polynomial function).

The median (IQR) observation time was 19 (12–31) months, during which 18.8% of participants discontinued treatment. At the end of the on‐drug period, 51.2% of patients had reached the maximum dose of 14 mg, while 46.1% and 2.7% remained on the 7 mg and 3 mg doses, respectively (Figure 1A). The estimated exposure is shown in Figure 1B, which illustrates inter‐individual variability resulting in some overlap in the *eC*_*avg*_ associated with prescribed doses in different patients over time.

Considering only the on‐treatment period, HbA1c declined by −0.7% (95% C.I. from −0.9% to −0.5%) at 12–18 months from a mean baseline of 8.0%, and then stabilised (Figure 1C). Body weight declined progressively from a baseline of 84.6 kg, reaching a maximum −7.3% (95% C.I. from −9.5% to −5.1%) at Year 4 (Figure 1D). GI side effects were reported at least once during observation in 41.8% of participants (Figure 1A).

### 3.3 Exposure and Glycaemic Improvement

**FIGURE 2:**
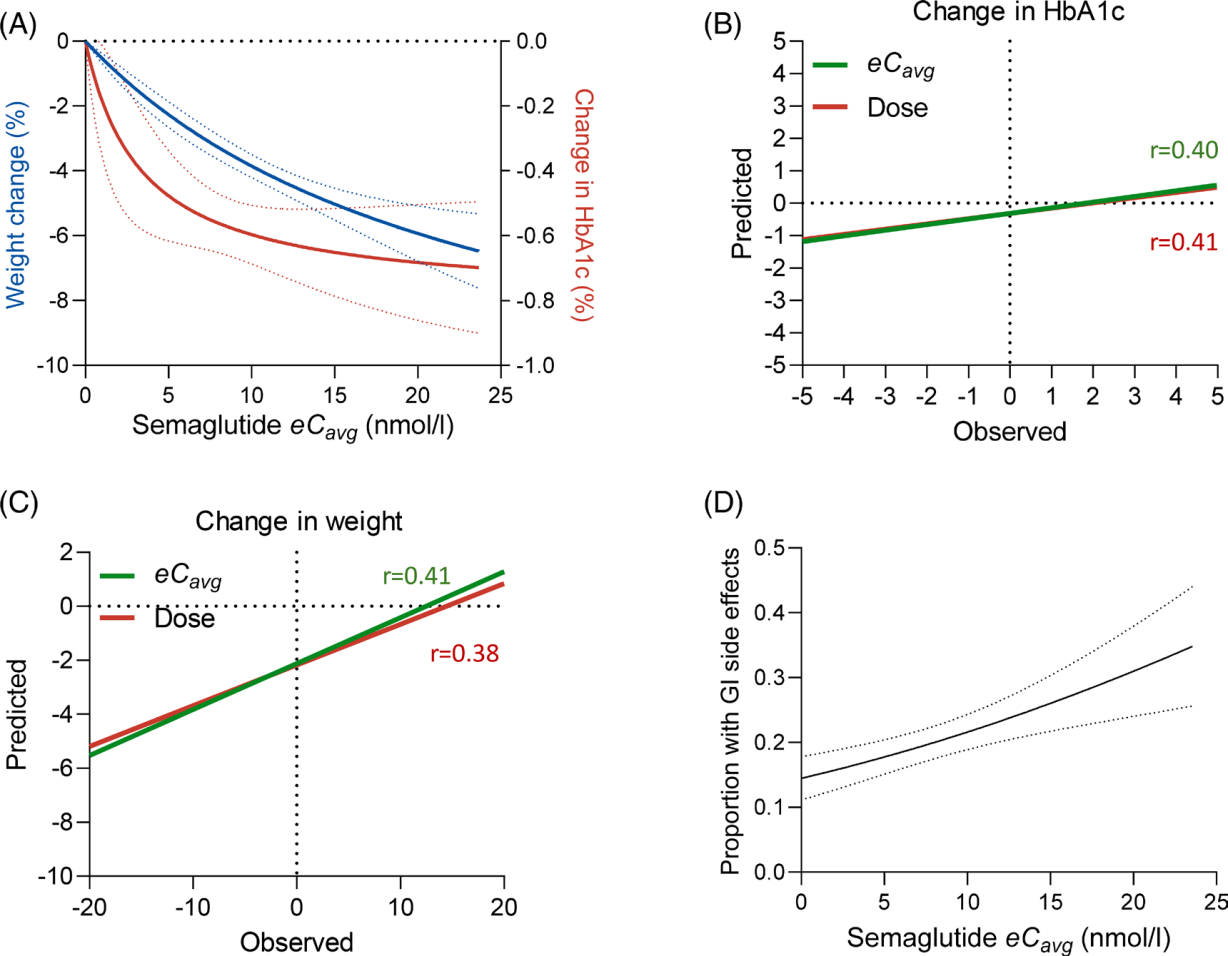
Modelled effects of exposure on the outcomes. (A) Modelled *E*_*max*_ relationship between exposure (estimated average concentration, *eC*_*avg*_) and change in HbA1c (red) or body weight (blue). Light dotted lines represent 95% confidence intervals. (B, C) Correlation between observed change in HbA1c (B) and percent body weight (C) and change in HbA1c or weight predicted by prescribed dose (red) or *eC*_*avg*_ (green). Correlation coefficients were not significantly different. (D) Modelled relationship between exposure (*eC*_*avg*_) and the proportion of participants with gastrointestinal (GI) side effects.

Considering all timepoints of all patients together, we modelled an *E*_*max*_ relationship between *eC*_*avg*_ and ΔHbA1c with a ceiling effect of −0.8% HbA1c reduction (95% from −0.5% to −1.5%) and an *EC*_*50*_ (concentration needed to obtain half of the maximum effect) equal to 3.4 nmol/L, with an upper limit of the 95% C.I. up to 15.8 nmol/L (Figure 2A).

To account for the interdependency of multiple observations in the same patients and model the relation between dose and exposure, we used mixed‐effects models. Both *eC*_*avg*_ (B = −0.039 ± 0.0056; *p* < 0.001; −2LL = 2501) and dose (B = −0.052 ± 0.0065; *p* < 0.001; −2LL = 2487) were significantly associated with greater reduction in HbA1c when entered individually in the mixed‐effect model. Due to the high correlation between dose and *eC*_*avg*_ (r^2^ = 0.93), they could not be included in the same model. Therefore, we compared the predictive performance of models including either dose or *eC*_*avg*_ alone. The model with dose showed a significantly better fit (absolute Δ−2LL = 14; *p* < 0.01), indicating that the prescribed dose was a more informative predictor of glycaemic response than the estimated exposure. In a mixed‐effects model including both *eC*_*avg*_ and the component of dose not correlated with *eC*_*avg*_ (dose residuals), both predictors remained independently associated with ΔHbA1c (*eC*_*avg*_ B = −0.026 ± 0.0057; *p* < 0.001; dose residuals B = −0.125 ± 0.0025; *p* < 0.001), with a marginal improvement in model performance compared to that with dose alone (absolute Δ−2LL = 4; *p* = 0.045). The correlation between observed and predicted values was similar for both models (*r* = 0.41 for dose vs. *r* = 0.40 for *eC*_*avg*_, *p* = 0.83 for the difference; Figure 2B), indicating that both dose and *eC*_*avg*_ yielded clinically comparable predictions of glycaemic response, despite the statistically better fit of the model with dose alone.

Finally, to account for the effect of drop‐in therapies, we performed a sub‐analysis where patients were censored at the time a new class of diabetes medications was added. The observed reduction in HbA1c was greater than in the primary analysis (−0.85%) and the dose model retained better performance compared with the *eC*_*avg*_ model (not shown).

### 3.4 Exposure and Weight Loss

In the analysis considering all time points of all patients, the observed relationship between *eC*_*avg*_ and percentage weight loss did not reach a clear plateau. The *E*_*max*_ (−12.9%) extended beyond the observed range, with an *EC*_*50*_ of 23.5 nmol/L (Figure 2A). These findings indicate that higher concentrations are needed to achieve meaningful weight reduction compared to HbA1c improvement.

In the mixed‐effect models accounting for interdependency of the observations, both *eC*_*avg*_ (B = −0.323 ± 0.022; *p* < 0.001; −2LL = 4831) and dose (B = −0.361 ± 0.026; *p* < 0.001; −2LL = 4850) were associated with greater weight loss when considered individually. Comparison of model fit confirmed the improved performance of the *eC*_*avg*_ model (absolute Δ−2LL = 19, *p* < 0.001). The correlation coefficient between observed and predicted percent weight change was significantly greater for *eC*_*avg*_ than for dose (*r* = 0.41 vs. 0.38, respectively; *p* = 0.022; Figure 2C).

*eC*_*avg*_ remained independently associated with the outcome in the mixed‐effects model independently from the dose residual (B = −0.331 ± 0.023; *p* < 0.001) without further improvement in model performance (absolute Δ−2LL = 1.4; *p* = 0.24), consistent with an exposure‐driven pharmacological effect.

### 3.5 Exposure and Occurrence of GI Side Effects

In the fitted curve considering all time points of all patients, the proportion of those with GI side effects tended to increase without achieving a well‐defined plateau, and the *EC*_*50*_ was 36.4 nmol/L (Figure 2D). In a mixed model for repeated measures accounting for time and time‐invariant patient characteristics, both *eC*_*avg*_ (B = 0.008 ± 0.003; *p* = 0.002; −2LL = 698) and dose (B = 0.010 ± 0.003; *p* = 0.002; −2LL = 698) had a positive correlation with GI adverse events with very similar model performance (*p* = 0.47). However, *eC*_*avg*_ (B = 0.008 ± 0.003; *p* = 0.002) retained a direct association with GI adverse events when entered in the model together with dose residuals, which lost significance (B = 0.007 ± 0.01; *p* = 0.541), suggesting that the estimated exposure provides more information over the dose in predicting GI tolerability.

## 4 Discussion

In this cohort of patients with T2D initiating oral semaglutide, we found that the relationship between drug exposure and clinical outcomes is more complex than what can be captured by the prescribed dose alone. For glycaemic control, the prescribed dose seems to be a more informative predictor, whereas for weight loss and GI tolerability, the estimated exposure provided greater predictive value and remained independently associated with the outcome even after adjusting for the component of dose not correlated with exposure.

The observation that the prescribed dose outperforms *eC*_*avg*_ in predicting HbA1c reduction (albeit by a small margin and based only on model fit) may reflect the fact that glycaemic improvement is a well‐established, dose‐dependent effect that is less influenced by inter‐individual differences in drug absorption. In clinical trials, the dose–response for HbA1c is steep at lower doses and plateaus at higher exposures [13], a pattern we also observed. The high correlation between dose and *eC*_*avg*_ makes it difficult to disentangle their effects, but the likelihood‐ratio test suggests that dose captures the majority of the information needed to predict glycaemic response. The HbA1c decrease we observed (estimated *E*_*max*_ −0.8%) is lower than the maximum effect of oral semaglutide in phase III trials (up to −1.4%) [14]. The possible explanations include as follows: (i) a lower baseline HbA1c in our cohort; (ii) the suboptimal dose titration, with a high proportion of patients not escalating to the 14 mg dose and some remaining on the 3 mg dose; (iii) limited compliance with pre‐ and post‐dose fasting under free‐living conditions may have reduced effectiveness. By contrast, the observed effectiveness on HbA1c, which was greater after censoring at therapeutic intensification, is in line with PIONEER‐Real, a series of country‐specific prospective real‐world studies on oral semaglutide [15, 16].

Our data confirm a well‐recognised principle of GLP‐1 receptor agonism: the concentration‐response curve for weight loss is shifted to the right compared to that for HbA1c [13, 17]. While the exposure‐response model for HbA1c reached a plateau with an *EC*_*50*_ of 3.4 nmol/L, the curve for body weight did not plateau. As the estimated *E*_*max*_ and *EC*_*50*_ for weight loss lie beyond the observed exposure range, they should be interpreted with caution, as parameter uncertainty is increased when a plateau is not directly observed. Nonetheless, this finding is entirely consistent with the results of recent trials exploring higher doses of oral semaglutide. In the OASIS 1 and 4 [18, 19] and PIONEER PLUS studies [12], doses of 25 mg and 50 mg daily produced additional weight loss compared to the 14 mg dose, without a commensurate further reduction in HbA1c, illustrating the right‐shifted nature of the dose–response for body weight.

The notion that *eC*_*avg*_ provided complementary information over dose for weight loss is substantiated by as follows: (i) the Δ−2LL test, the effect of *eC*_*avg*_ independent from dose residuals; (ii) the more robust correlation between observed outcome values and the values predicted from *eC*_*avg*_ than from prescribed dose, although the absolute difference in correlation coefficients was small and may have limited clinical relevance.

This finding can be explained at least in part by a bidirectional relationship between exposure and body weight. Weight per se is a determinant of *eC*_*avg*_ in the pharmacokinetic model [7]. As patients lose weight, the same prescribed dose results in higher plasma concentrations, creating a positive feedback loop that is captured by the estimated exposure more than by the dose alone. In other words, *eC*_*avg*_ integrates not only the prescribed dose but also the individual's changing weight, making it a more dynamic and informative metric for weight‐related outcomes.

The independent association of *eC*_*avg*_ with GI side effects, even after adjusting for the dose residual, highlights the importance of inter‐individual variability in drug absorption for tolerability. The overlap in *eC*_*avg*_ achieved with different doses means that a patient on 7 mg semaglutide may have a comparable exposure to another on 14 mg, and consequently a similar risk of side effects. This finding suggests that the estimated exposure may be a more reliable predictor of tolerability than the prescribed dose, and that clinicians should be aware of the factors that influence an individual's semaglutide pharmacokinetic profile.

Although statistical superiority does not necessarily imply greater clinical utility, particularly given the modest magnitude of the observed differences, our findings may have practical implications. First, we highlight the importance of considering inter‐individual variability in exposure when prescribing oral semaglutide. The fact that the same dose can yield widely different concentrations at steady state in different patients [7], and that these concentrations predict weight loss and GI tolerability better than the dose itself, suggests that a personalised approach guided by simple clinical characteristics could optimise outcomes. Whether this will hold true for future oral GLP‐1RA, such as orforglipron [20, 21], remains to be determined, as small molecule agents usually show less variable absorption profiles. Second, our study raises the question of whether it is worth estimating exposure upfront to guide clinical decisions. Direct measurement of semaglutide concentrations is not available outside specialised research settings and industry‐sponsored studies, limiting the feasibility of such approaches in real‐world studies. Rather, the use of a validated pharmacokinetic model incorporating easily available variables provides a practical tool to estimate an individual's exposure. This could help identify patients who are at risk of achieving sub‐therapeutic concentrations or, conversely, those who may be more prone to side effects at a given dose, allowing for more informed dose titration.

The main strengths of this study include its real‐world design, which provides insights complementary to those obtained from trials. We enrolled a well‐characterised cohort with extensive data on clinical characteristics, concomitant medications and longitudinal outcomes. The application of a published pharmacokinetic model to estimate individual exposure [7], and the use of rigorous mixed‐effects models to account for the longitudinal structure of the data and to compare the predictive value of dose and exposure, represent methodological strengths. To our knowledge, this is the first study to directly compare the predictive value of estimated exposure versus prescribed dose for multiple outcomes in a real‐world cohort of patients treated with oral semaglutide. Our findings are broadly consistent with those of Overgaard and colleagues [7], who showed, using data from the PIONEER and SUSTAIN trials, that circulating semaglutide levels determine reductions in HbA1c and body weight, irrespective of the route of administration. The present work extends these observations in a real‐world setting with its inherent variability in adherence and clinical characteristics.

Several limitations should be acknowledged. First and most important, exposure was estimated using a published pharmacokinetic model rather than directly obtained from measured semaglutide concentrations. Direct measurement would require specialised bioanalytical assays and prospectively collected plasma samples, which are not available in retrospective real‐world clinical cohorts. Therefore, direct pharmacokinetic validation was not feasible in the present independent observational study. As our cohort may be significantly different from patients in the PIONEER trial programme, wherein the *eC*_*avg*_ model was developed, it remains uncertain whether the model performance is preserved in a real‐world setting. In addition, incomplete adherence, missed doses, dose holidays and deviations from administration instructions could lead to exposure misclassification, the direction and magnitude of which cannot be determined in the present study. Also, we note that body weight is included in the pharmacokinetic model, driving a partial dependency between exposure and outcome and potentially contributing to the observed association with weight loss. Sex, which is a determinant of GLP‐1RA effects [22], is also incorporated in the *eC*_*avg*_ equation, but women were under‐represented in our cohort. It should be mentioned that dose escalation was incomplete in many patients and the reasons for not reaching higher doses were not captured and are often hard to understand in retrospective studies. As exposure was estimated at each visit based on the current dose, it reflects real‐time dose adjustments, but treatment discontinuation may introduce selection bias, as patients experiencing adverse events may be underrepresented at later time points. In addition, we estimated *C*_*avg*_ assuming steady‐state and not the *C*_*max*_, which may be more relevant for GI tolerability [23]. Second, this is a single‐centre study, which may limit the generalisability of our findings to other populations or healthcare settings. Third, the absence of a placebo or active comparator arm means that we cannot establish causality, only associations. Finally, the retrospective design cannot exclude the possibility of residual confounding by unmeasured variables, such as dietary habits or physical activity, which may influence both weight loss and glycaemic control. GI adverse events were assessed retrospectively using chart‐derived terms mapped to standard MedDRA categories and analysed as a binary outcome, without detailed information on severity or symptom type, which may limit the granularity of the analysis.

In conclusion, this real‐world study suggests that the relationship between estimated exposure to oral semaglutide and clinical outcomes is multifaceted: while the glycaemic effect may be predicted by the prescribed dose alone, weight response and GI tolerability appear to depend more on exposure. These findings may support the use of simple pharmacokinetic models to complement clinical judgement when initiating and titrating oral semaglutide. Future studies should investigate whether prospective use of exposure estimates can improve clinical outcomes.

## Author Contributions

G.P.F. designed the study, analysed data and wrote the manuscript. M.L.M. researched and analysed data and revised the manuscript. B.M.B. and C.B. researched data and revised the manuscript. A.C. designed the study and revised the manuscript. All authors contributed significantly to the work and approved the final version of the manuscript.

## Funding

Supported by the Università degli Studi di Padova.

## Conflicts of Interest

G.P.F. received consultancy, advisory board or lecture fees from AstraZeneca, Boehringer, Lilly, Guidotti, Sanofi, Novo Nordisk, Novartis, Servier. M.L.M. received consultancy, advisory board or lecture fees from AstraZeneca, Boehringer, Guidotti, Novartis, Novo Nordisk, Sanofi and Servier. B.M.B. received lecture or advisory board fees from AstraZeneca, Boehringer Ingelheim, Lilly, M.S.D., Sanofi, Servier and Novo Nordisk. A.C. received lecture fees from Servier and Recordati. C.B. has nothing to declare.

## Data Availability

Restrictions apply to sharing original data used for this study. Aggregate data are available from the corresponding author upon reasonable request.
